# Development and Application of an RT-PCR to Differentiate the Prevalent NA-PRRSV Strains in China

**DOI:** 10.2174/1874357901711010066

**Published:** 2017-06-30

**Authors:** Yanlin Li, Guobiao Ji, Xiaodong Xu, Juan Wang, Yingying Li, Feifei Tan, Xiangdong Li

**Affiliations:** 1 College of Animal Science and Veterinary Medicine, Henan Agricultural University, Zhengzhou, China; 2 National Research Center for Veterinary Medicine, Luoyang, China

**Keywords:** PRRSV, North American genotype, HP-PRRSV, NADC30-like PRRSV, RT-PCR

## Abstract

**Background:**

PRRSV features with genetic diversity and high mutation which leads to the emergence of a multiple of circulating virus strains with different virulence. North American (genotype 2) PRRSV (NA-PRRSV) can be divided into classical PRRSV (C-PRRSV), highly pathogenic PRRSV (HP-PRRSV), and NADC30-like PRRSV (NL-PRRSV) according to their genomic characteristics and pathogenicity. So far, the above three subtypes of NA-PRRSV are now circulating in China.

**Objective and Method:**

In this study, a reverse transcript polymerase chain reaction (RT-PCR) was established to simultaneously differentiate three subtypes of NA-PRRSV. The established RT-PCR can be applied to PRRSV-infected samples originated from both supernatant of cell culture and pig tissues and showed specificity exclusively to PRRSV. The sensitivity of RT-PCR showed the minimum RNA detection was 0.04ng/µl.

**Result and Conclusion:**

The established RT-PCR was next used to differentiate the subtypes of 29 NA-PRRSV isolated in 2016 and the results showed that HP-PRRSV is still the dominant circulating virus strain in the presence of NADC30-like PRRSV in Henan province.

## 1 INTRODUCTION

PRRS is one of the most economically important swine diseases worldwide since last century. The etiologic agent, PRRSV, belongs to the order *Nidovirales*, family *Arteriviridae*. PRRSV can be divided into European genotype (type 1) and North American genotype (type 2) with Lelystad and VR-2332 as prototypical strains, respectively [1]. Within each genotype, the viruses could be divided different subtypes according to their genomic characters and pathogenicity [2]. In China, the first NA-PRRSV was reported in 1996 and became endemic nationwide since then [3]. HP-PRRSV emerged in 2006 with genetic marker of two discontinuous 30aa (29aa+1aa) deletion in NSP2 gene [4]. HP-PRRSV led to the death of more than 1 million pigs with all ages merely in 2006 and had become the dominating PRRSV strain in Asia [4]. Recently, the emergence of NADC30-like PRRSV in China that showed the highest similarity to a group represented by NADC30, a type 2 PRRSV that has been isolated in the United States of America in 2008, draw much attention from veterinarian due to the outbreaks of it from vaccinated pig herds [5]. NL-PRRSV has a unique three discontinuous 131aa deletion (111a+1aa+19aa) in NSP2 gene which could distinguish themselves with other PRRSV strains [6].

Coexistence of above three subtypes of NA-PRRSV in pig herds makes the disease control more difficult in the field. It is urgent to develop a rapid and sensitive method such as polymerase chain reaction (PCR) to differentiate different subtypes of PRRSV. Therefore, in this study, we established an RT-PCR with high specificity and sensitivity to differentiate these viruses which can be applied to both cell culture and pig tissues.

## 2 MATERIALS AND METHODS

### 2.1 Viruses and Cells

PRRSV strains VR-2332 and JXA1 were maintained in National Research Center for Veterinary Medicine and propagated in Marc145 cells. An NADC30-like strain, HNjz15, was propagated in porcine alveolar macrophages (PAMs) as previously described [7].

### 2.2 Primer Design

According to the representative NSP2 genes of reference PRRSV strains Fig. (**1**), a pair of primers was designed to differentiate three subtypes of NA-PRRSV: forward, 5’-TTGATTGGGATGTTG TGCTTC-3’, and reverse, 5’-CAATGATGGCTTGAGCTGAGT-3’ with expected 1074bp, 984bp, and 681bp gene fragments for C-PRRSV, HP-PRRSV, and NL-PRRSV, respectively.

### 2.3 RNA Extraction and RT-PCR Amplification

The viral RNAs were extracted from lung tissues and cell cultures using TRIzol reagent as previously described [8]. One microgram of RNA from each sample was used to synthesize cDNA using Oligo (dT)_18_ primer (Life Technologies, Beijing, China). Reverse transcription was performed by adding 1µL reverse transcriptase (TaKaRa, Dalian, China), 0.5µL recombinant rebonuclease inhibitor (40U/µL), 4µL RT buffer and 2ul dNTPs (10µM) at 42°C for 1hour. For PCR amplification, 2.5µL 10×buffer, 2µL of 10µM dNTPs, 1µL of each primer (10µM), and 0.2µL Taq polymerase (TaKaRa, Dalian, China), and 0.1µg cDNA was added into the reaction by adding deionized H_2_O to a final volume of 25µL. PCR was performed using following parameters: denaturation at 94 °C for 5 min, 30 cycles of denaturation at 94°C for 30 s, annealing at 55 °C for 30s and extension at 72 °C for 45s, and a final extension of 7 min at 72 °C. The PCR products were analyzed by 2% agarose gel electrophoresis.

### 2.4 Specificity of RT-PCR

The specificity of RT-PCR was used to detect three different subtypes of NA-PRRSV in both cell cultures and lung tissues which infected with VR-2332, JXA1, and HNjz15, respectively. To exclude cross-reaction with other swine pathogens, total RNA of pseudorabies virus, porcine epidemic diarrhea virus, classical swine fever virus, and transmissible gastroenteritis virus was used to perform RT-PCR. The above swine viruses were maintained at National Research Center for Veterinary Medicine.

### 2.5 Sensitivity of RT-PCR

*The cDNA was synthesized from* 1µg total RNA of three subtypes of NA-PRRSV, respectively. *The cDNA* was then serially ten-fold diluted and used as template for PCR. The amplification parameters were same as above. The sensitivity was expressed the maximum cDNA dilution which the gene fragments was still visible on 2% agarose gel.

### 2.6 Utilization of RT-PCR on Clinical Samples

Twenty-nine PRRSV positive serum samples were tested by using the above developed RT-PCR (Table **1**). The serum samples were collected form the PRRSV vaccination- free herds. The established RT-PCR was used to differentiate subtypes of above NA-PRRSV.

## 3 RESULTS

### 3.1 Establishment of RT-PCR

The total RNA of cell cultures infected with different PRRSV strains were extracted to be used as template for RT-PCR. According to amplicons of RT-PCR, the fragment sizes of PCR products were 1074bp, 984bp, and 681bp gene fragments for C-PRRSV, HP-PRRSV, and NL-PRRSV, respectively Fig. (**2**). As for the negative control, no visible band was found on the gel from normal cell cultures without virus infection. The PCR products were subjected to gene sequencing and the results showed that they shared the same sequences with corresponding reference virus strains (data not shown).

### 3.2 Specificity of RT-PCR

**Fig. (3):**
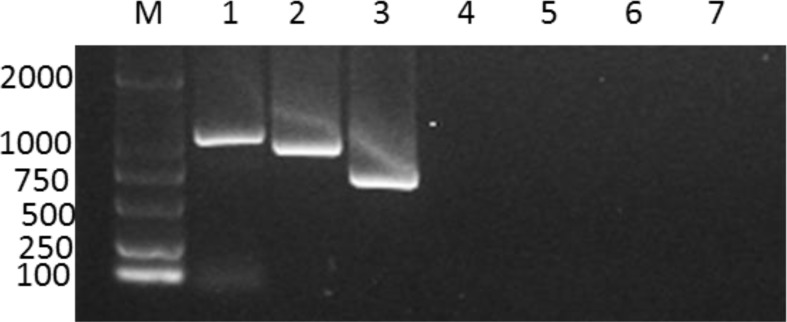
**RT-PCR products from the infected tissue samples.** Line1, DL2000 DNA ladder; line 2-4, PCR product of VR-2332, JXA1, and HNjz15; Line 4-7, PCR products of pseudorabies virus, porcine epidemic diarrhea virus, classical swine fever virus, and transmissible gastroenteritis virus.

To explore the specificity of RT-PCR, total RNA extracted from PRRSV-infected lung tissues was used as template for the PCR reaction. Consistent with RT-PCR results from cell culture, similar size of gene fragments was visible for each PRRSV on the gel which proved the established method could be applied to lung tissues Fig. (**3**). Next, the developed RT-PCR was further tested with pseudorabies virus, porcine epidemic diarrhea virus, classical swine fever virus, and transmissible gastroenteritis virus and no cross-reaction with above pig pathogens was observed (Fig. **3**).

### 3.3 Sensitivity of RT-PCR

CDNAs were serially diluted and used as template to validate sensitivity of RT-CPR. As shown in Fig. (**4**), the minimum detection amount of RNA was 0.04ng/µL for VR-2332, JXA1 and HNjz15.

### 3.4 Application of Established RT-PCR on Clinical Samples

Twenty-nine NA-PRRSV-positive serum samples were next used to differentiate the subtypes of NA-PRRSV (Table **1**). Among these samples, 26 (89.6%) samples were positive for HP-PRRSV and 3 samples for NL-PRRSV. There was no C-PRRSV detected from above samples.

## 4 DISCUSSION

PRRSV features with rapid evolution with gene mutation and recombination which lead to the emergence of genetically and antigenically heterogeneous population [9, 10]. So far, three subtypes of NA-PRRSV including C-PRRSV, HP-PRRSV, and NL-PRRSV are co-circulating in China which make the situation of PRRSV control challenging. Vaccination has been used as one the major measurements to control this disease in China [11]. There are six commercial PRRSV modified live vaccines that have been widely used in China.

Among these six vaccines, four vaccine strains originated from HP-PRRSV, and two vaccines originated from C-PRRSV. No commercial vaccines again NL-PRRSV is available. Since the cross-protection of above vaccines to different subtypes of PRRSV is limited, it is prerequisite to differentiate subtypes of viruses from the infected pigs before performing vaccination or other exigent measurements.

PRRSV NSP2 is the most variable gene that undergoes remarkable genetic variations which makes it the main target for phylogenetic analysis [12]. The full length of C-PRRSV represented by VR-2332 is 1196aa without any deletion. HP-PRRSV has a 30aa discontinuous deletion which consists of 1-aa deletion at position 482 and 29-aa deletions at 534-562 [4]. By contrast, NL-PRRSV has a three discontinuous 131-aa deletion which consists of a 111-aa at position 323-433, a 1-aa deletion at position 481, and a 19-aa deletion at position 533-551 Fig. (**1**) [6]. The above distinct deletion patterns make it possible to differentiate three subtypes of NA-PRRSV simply according to the sizes of PCR products.

The RT-PCR was firstly established by using RNA extracts from PRRSV-infected cells. The desired gene fragments were observed on 2% agarose gel. The sequencing results of PCR products proved the successful establishment of RT-PCR. Since the virus in cells culture is homogenous, we next tested the established RT-PCR by using RNA extracts from PRRSV-infected lung tissues. The results also showed that the RT-PCR is compatible with tissue-originated RNAs. To test the specificity of RT-PCR, the assay was used to detect several other pig viral pathogens, and no target bands were detected on the gel.

Viremia starts as early as 3 days post-infection of PRRSV and lasts for around one month with different virus titers in serum at different stages of virus infection [10]. After that, virus titer is pretty low in blood and the residues of PRRSV are mainly found in lung and lymphoid tissues such as lymph nodes and tonsil. Therefore, the sensitivity of RT-PCR is critical to detect the virus. In this study, the minimum detected virus RNA was 0.04ng/µl for all three subtypes of NA-PRRSV which could be sensitive enough to detect RNA residue in the virus-infected tissues.

HP-PRRSV has been dominantly circulating virus strain in China since 2006 [2]. The emergence of PRRSV variants with different virulence and pathogenicity was constantly reported recently [6, 7]. One representative of these variants is NADC30-like PRRSV that was reported in 2015 [13]. So far, 11 NADC30-like PRRSV genome information is available at Genbank.

Among these 11 NADC30-like PRRSV, 7 of them were isolated in Henan province, Central China which indicates Henan province has been the endemic area for NL-PRRSV. In this study, 29 PRRSV-positive serum samples were collected in 2016 and were used to differentiate the subtypes of NA-PRRSV. Three (10.4%) of these PRRSV-positive serum samples were verified to be NADC30-like PRRSV infection which indicated the high incidence of this PRRSV variant infection in local pig farms.

## CONCLUSION

In conclusion, an RT-PCR was established which can differentiate classical, highly pathogenic, and NADC30-like PRRSVs with good specificity and sensitivity. Epidemiological survey of PRRSV performed in Henan province showed NADC30-like PRRSV accounting for around 10% of PRRSV isolates in first half year of 2016.

## Figures and Tables

**Fig. (1):**
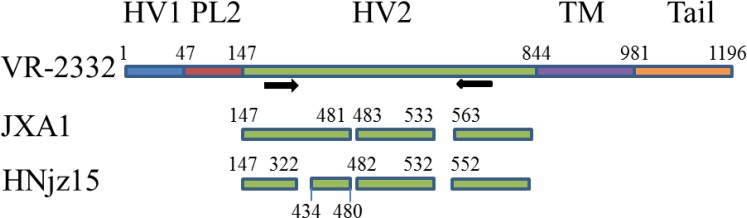
**Representative Deletions in PRRSV NSP2 gene.** PRRSV JXA1 and HNjz15 were compared with stereotypical NA-PRRSV VR-2332. Five different function regions of NSP2 gene were indicated by different colors and marked according to the amino acid location in NSP2. Black arrows indicated the location on NSP2 where primers were designed. Abbreviation: Hypervariable region 1(HV1), PLP2 cysteine protease core (PL2), Hypervariable region 2 (HV2), Transmembrane region (TM), C-terminal tail (Tail).

**Fig. (2):**
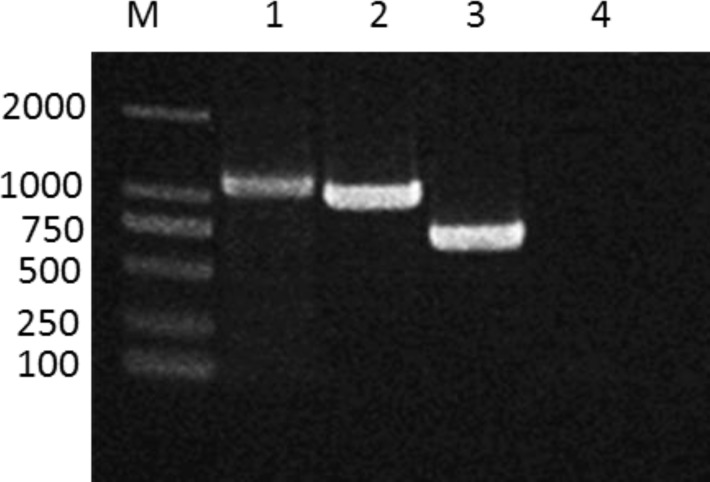
**RT-PCR products of PRRSV from infected cell lines.** Line1, DL2000 DNA ladder; line 2, PCR product of VR-2332-infected cells; PCR product of JXA1-infected cells; PCR product of HNjz15-infected cells; PCR product of normal cells.

**Fig. (4):**
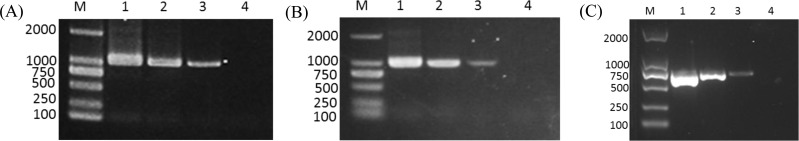
**Sensitivity of established RT-PCR.** Total RNA of VR-2332 (A), JXA1 (B) and HNjz15 (C) were isolated from the infected cells. One microgram RNA was reverse-transcribed and cDNA were serially (10×) diluted to be used as the template for PCR amplification. The bottom limit of RT-PCR detection was expressed by the visualized bands on 2% agarose gel electrophoresis.

**Table 1:** Information about the viruses used in this study.

**No.**	**Virus Code**	**Subtype**	**Isolated From**
1	16105-1	HP-PRRSV	Henan
2	16124-1	HP-PRRSV	Shandong
3	16124-3	HP-PRRSV	Shandong
4	16124-5	HP-PRRSV	Shandong
5	16124-6	HP-PRRSV	Shandong
6	16213-1	NADC30-like	Henan
7	16213-2	NADC30-like	Henan
8	16214-1	HP-PRRSV	Shanxi
9	16214-2	HP-PRRSV	Shanxi
10	16214-3	HP-PRRSV	Shanxi
11	16214-4	HP-PRRSV	Shanxi
12	16224-1	HP-PRRSV	Henan
13	1693-7	HP-PRRSV	Jiangsu
14	16690-1	HP-PRRSV	Gansu
15	16319-21	HP-PRRSV	Shanxi
16	16319-23	HP-PRRSV	Shanxi
17	16319-25	HP-PRRSV	Shanxi
18	16319-32	HP-PRRSV	Shanxi
19	16319-33	HP-PRRSV	Shanxi
20	16319-35	HP-PRRSV	Shanxi
21	16319-37	HP-PRRSV	Shanxi
22	16346-1	HP-PRRSV	Shanxi
23	16354-2	HP-PRRSV	Tianjin
24	16354-12	HP-PRRSV	Tianjin
25	16494-1	HP-PRRSV	Shanxi
26	16494-3	HP-PRRSV	Shanxi
27	16494-4	HP-PRRSV	Shanxi
28	16628-1	HP-PRRSV	Henan
29	16756-2	NADC30-like	Henan
